# Time-updated explainable machine learning predicts short-term mortality in peritoneal dialysis patients

**DOI:** 10.1080/0886022X.2026.2666955

**Published:** 2026-05-27

**Authors:** Quan Wang, Yanqiong Ding, Qing Luo, Sheng Wan, Yanmin Zhang

**Affiliations:** Department of Nephrology, Wuhan No.1 Hospital, Wuhan, China

**Keywords:** Continuous ambulatory peritoneal dialysis, mortality risk prediction, explainable artificial intelligence, machine learning models, electronic health records

## Abstract

**Objective:** This study aimed to develop and validate a time-updated, explainable machine learning (ML) early-warning system for short-term mortality risk in patients with continuous ambulatory peritoneal dialysis (CAPD). **Methods:** Multiple supervised ML techniques were validated to stratify individuals at high risk of 6-month death, classifiers were trained, internally validated and independently temporally validated in this retrospective study of 1,484 CAPD patients in our PD center. We collected time-updated markers such as patient demographics, clinical characteristics, and laboratory data to inform the ML models and the performance of eight ML models was assessed *via* the area under the curve (AUC) and accuracy. The SHapley Additive exPlanation (SHAP) method was selected to further interpret the predictive models and link findings to clinical actionability. **Results:** In internal validation cohort, the light gradient boosting machine (lightGBM) model demonstrated best performance with AUC of 0.888 and highest accuracy of 0.879. In temporal validation cohort, the lightGBM model also exhibited good classification performance, achieving AUC of 0.850 and good accuracy of 0.874. Moreover, SHAP analysis identified several key features that contributed to the accurate prediction of the lightGBM model. **Conclusions:** The time-updated lightGBM early-warning system helps clinicians to early identify CAPD patients with high risk of death within six months based on easily accessible and time-updated data and provides a basis for personalized treatment.

## KEY CLINICAL TAKEAWAYS

1. We developed and validated a time-updated, explainable ML early-warning system that accurately predicts 6-month all-cause mortality in CAPD patients using routinely, available laboratory data.
2. The LightGBM model achieved excellent discriminative performance, with an AUC of 0.888 in internal validation and 0.850 in independent temporal validation.
3. This model provides a scalable, low-cost early-warning tool for routine CAPD care, with a standardized risk-stratified intervention framework to guide timely, targeted care and advance care planning.

## Introduction

Continuous ambulatory peritoneal dialysis (CAPD) is a widely used therapy for end-stage kidney disease (ESKD) patients, offering benefits such as preservation of residual renal function and improved quality of life [1,2]. However, CAPD patients remain at high risk for mortality, particularly from cardiovascular causes, with reported rates ranging from 15% to 40% over 3–5 years [3,4]. Therefore, early identification of high-risk patients is crucial for timely intervention and personalized care.

Conventional risk prediction models in CAPD rely on clinical characteristics in a specific time, especially laboratory results and imaging indices [5,6]. Beyond the relatively high medical cost of specialized testing, these approaches often lack integration of multi-dimensional and time-updated EHR data and may not fully capture complex interactions among risk factors. In addition, existing models typically demonstrate moderate predictive accuracy, leaving room for improvement in clinical utility. Moreover, while many studies specialized in longer-term mortality, there is still crucial in administering patients might die sooner (less than 1 year), due to the variability of death rates and medical decision-making [7,8].

Machine learning (ML) methods have revolutionized prognostic modeling across medical specialties by efficiently handling high-dimensional data and identifying complex, non-linear relationships [9,10]. In nephrology, ML applications have been widely performed [11] and shown promising results in various domains: predicting mortality in hemodialysis (HD) patients [12], forecasting CKD progression [13,14], and identifying acute kidney injury in hospitalized patients [15]. Despite these advances, the application of ML models for mortality prediction in CAPD populations remain limited [16]. The growing availability of EHR data in dialysis clinics provides an unprecedented opportunity to leverage ML approaches for comprehensive risk assessment in this specific population. Hence, in this study, we aim to develop and validate a time-updated, explainable ML early-warning system for predicting short-term mortality in CAPD patients using comprehensive and time-updated EHR data, which in turn will assist clinicians in identifying CAPD patients with high risk, and provide significant clue for the personalized treatment plans, ultimately improving the prognosis of CAPD patients.

## Methods

### Study design and population

We conducted a retrospective cohort study of CAPD patients treated at Wuhan No.1 Hospital between January 2021 and February 2025. Our dialysis clinic is one of the biggest in the middle of China, with over 400 PD patients and more than 700 HD patients getting treatment at our facility. Moreover, our dialysis clinic employed an electronic data collection system to gather patient-level data quarterly, which means that every patient had at least four times as many characteristics once a year if they did not die or were moved to HD or kidney transplantation.

**Figure 1.**
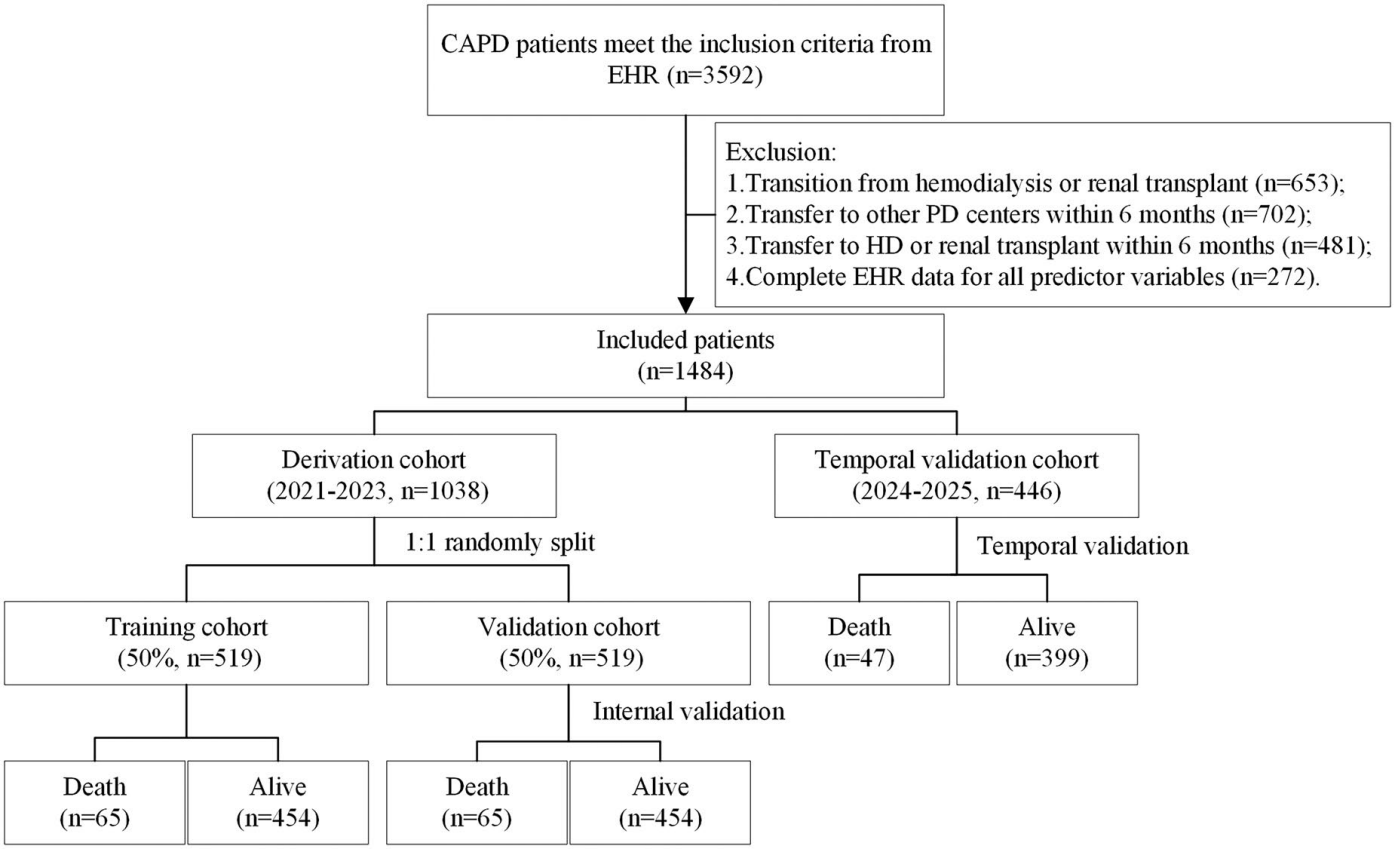
The flowchart of the patient selection.

Inclusion criteria were: (1) age ≥ 18 years, (2) CAPD as initial RRT for at least 3 months, and (3) availability of complete EHR data for all predictor variables. Exclusion criteria included: (1) transition from hemodialysis or failed renal transplant, (2) had missing or unclear EHR data, and (3) transfer to other dialysis centers within 6 months, and (4) transfer to HD or kidney transplantation within 6 months. Finally, this study comprised 1484 CAPD patients (Figure 1). Patients from 2021 to 2023 were formed the training cohort (50%, *n* = 519) for ML model development, and an internal validation cohort (50%) for internal validation (*n* = 519) using a patient-level clustered stratified random splitting strategy (detailed in the Supplemental Methods), while the data from 2024 to 2025 were used as temporal validation cohort for temporal validation (*n* = 446).

The Ethics Committee of Wuhan No. 1 Hospital approved this study (No. 2025-71), and considering that this was a retrospective study and all patients were de-identified or maintained with confidentiality, informed consent was waived by the Ethics Committee of Wuhan No. 1 Hospital.

### Data collection and preprocessing

In order to develop an easy-to-use prediction model and did not impose additional medical expenses on them, we only included characteristics that were easily available in the EHR database, such as demographics, dialysis duration, and laboratory findings. Furthermore, we arranged the data in a time-updated patient-semester manner, with one row of data for each calendar semester in which a patient attended at least one CAPD session at our center. Each routine quarterly laboratory assessment constituted a separate data point, linked to a subsequent 6-month outcome window for all-cause mortality. The prediction timeline was strictly defined as: (1) T0 (prediction start): the date of a patient’s routine quarterly laboratory test; (2) Outcome window: all-cause mortality within 6 months after T0. If a patient had multiple tests per semester, all were included separately, each associated with its own six-month risk period. A total of 42 variables were extracted from EHRs and the detailed features included in this study were listed in Table S1.

Considering that kidney transplantation is a competing event and that may prevent death on CAPD, we excluded patients who received transplantation within the 6-month outcome window, an accepted approach for competing event handling in CAPD mortality studies.

#### Definition of ‘independent observations’

Each ‘sample’ corresponds to a single routine quarterly laboratory test, with the outcome defined as all-cause mortality within 6 months after that specific test. For patients with multiple quarterly laboratory tests, each test is treated as an independent observation-because each test reflects the patient’s clinical status at a distinct time point, and the outcome (6-month mortality) is specific to the period following that test (not a global outcome for the patient). This design captures dynamic changes in clinical parameters and their association with short-term mortality, rather than treating multiple tests from the same patient as ‘repeated measures of the same outcome.’

#### Feature selection

As multi-collinearity between clinical variables might weaken the predictive ability of models, a rigorous three-step strategy was employed for feature dimensionality reduction and selection. Initially, low-variance features, which offer minimal discriminative power, were discarded. Subsequently, to mitigate redundancy, features exhibiting a high pairwise correlation (Spearman’s correlation coefficient > 0.8) were removed. Finally, since Boruta feature selection method is a useful tool for feature selection that identifies all relevant features [17], we utilized ‘Boruta’ R package to further select the important indexes. This two-stage pre-filtering followed by Boruta selection reduces computational burden, stabilizes feature importance estimation, and preserves clinically relevant signals.

### ML models construction and evaluation

ML models for 6-month mortality risk stratification were developed using the training dataset. Eight established ML algorithms were selected: Logistic Regression (LR), Random Forest (RF), k-Nearest Neighbors (KNeighbors), Decision Tree (DT), eXtreme Gradient Boosting (XGBoost), Light Gradient Boosting Machine (LightGBM), Support Vector Machine (SVM), and a Stacking ensemble classifier [18]. The Synthetic Minority Over-Sampling Technique (SMOTE) was applied to balance the dataset. Hyperparameter tuning was performed using 5-fold cluster cross-validation (clustered at the patient level) combined with a grid search in the training set, to account for within-patient correlation and prevent data leakage.

Model performance was assessed across the internal validation, and temporal validation cohorts. Performance metrics, including accuracy, sensitivity, specificity, and Brier score, were calculated. Discriminatory ability was evaluated using the area under the receiver operating characteristic curve (AUC). The Brier score was used as a quantitative measure of both discrimination and calibration, with lower scores indicating superior performance. Calibration plots were utilized to visually assess the agreement between model-predicted probabilities and actual observed outcomes. Decision curve analysis (DCA) was performed to quantify the net clinical benefit of the optimal model across clinically relevant risk thresholds.

### Feature importance and model interpretation

**Figure 2.**
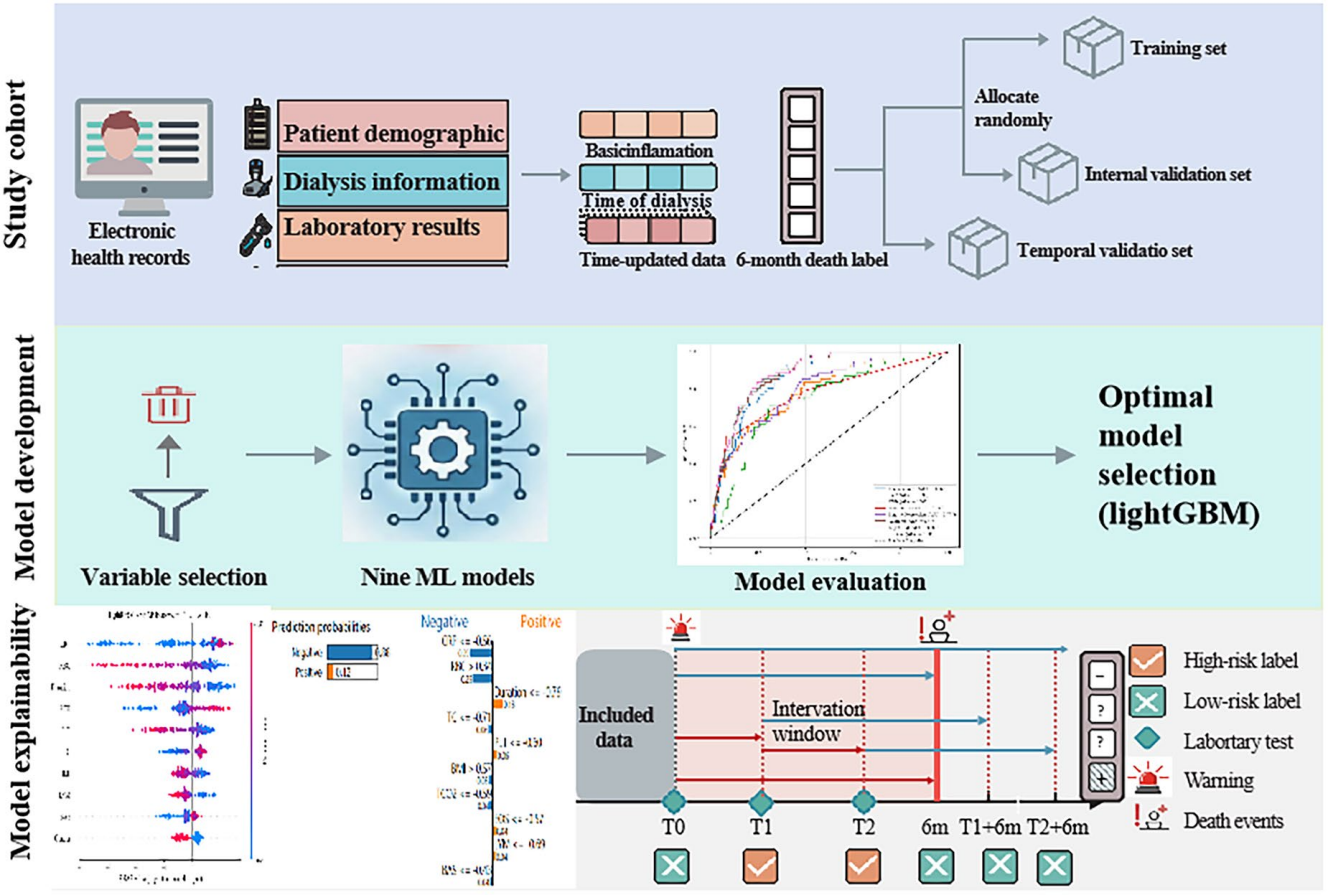
The graphical workflow of this study.

Given the inherent complexity of ML models, which can often function as ‘black boxes’, we employed SHapley Additive exPlanations (SHAP) to enhance the interpretability of the best-performing model. SHAP summary analysis provided global insight into the overall feature importance across the dataset. For local, instance-level explanations, Local Interpretable Model-agnostic Explanations (LIME) were implemented to elucidate the rationale behind individual predictions. Furthermore, SHAP dependence plots were generated for the top four most influential features to visualize how variations in each feature impact the model’s output. The top modifiable features identified by SHAP were explicitly linked to evidence-based clinical interventions to enhance the model’s clinical actionability. The graphical workflow of this study is shown in Figure 2.

### Statistical analysis

Baseline characteristics were compared using t-tests for normally distributed continuous variables, Mann-Whitney U tests for non-normal distributions, and chi-square tests for categorical variables. All analyses were performed in R (version 4.3.2) and Python (version 3.11). Detailed information on the analysis and feature selection are provided in the Supplementary materials. A two-sided p-value <0.05 was considered statistically significant.

## Results

### Baseline characteristics

The baseline characteristics of all patients had been described in the Table 1. The distributions of all features including the 6-month mortality, were comparable between the two cohorts. The mean age was 61 years, and the mortality rates were 12.5% and 10.6%, respectively, in both sets.

**Table 1.** Comparisons of baseline characteristics in all cohorts.

Characteristics	Derivation cohort	Temporal validation cohort	*P* value
*N*	1038	446	–
Age, years old	60.8 ± 13.2	61.2 ± 12.5	0.624
Sex, male, *n* (%)	582 (56.0)	257 (57.8)	0.536
BMI, kg/m^2^	22.8 ± 3.4	22.6 ± 3.3	0.505
Duration of CAPD, months	38.0 (20.0, 51.0)	42.0 (24.0, 61.0)	0.226
Laboratory values			
WBC, × 10^9^/L	6.8 ± 1.3	6.7 ± 2.5	0.113
Hemoglobin, g/dL	106.0 ± 20.9	107.1 ± 20.3	0.368
Platelet, × 10^9^/L	209.5 ± 61.4	212.7 ± 59.5	0.419
RBC, × 10^12^/L	3.7 ± 0.9	3.7 ± 0.7	0.811
Neutrophil, × 10^9^/L	4.6 ± 1.1	4.5 ± 1.2	0.119
Lymphocyte, × 10^9^/L	1.4 ± 0.4	1.4 ± 0.5	0.720
Monocyte, × 10^9^/L	0.45 ± 0.10	0.46 ± 0.12	0.973
Basophil, × 10^9^/L	0.03 ± 0.01	0.03 ± 0.01	0.924
Eosinophil, × 10^9^/L	0.27 ± 0.08	0.25 ± 0.06	0.746
MPV, fL	10.0 ± 1.5	10.0 ± 1.4	0.894
MCH, pg	28.2 ± 3.7	28.2 ± 3.8	0.693
MCHC, g/L	325.9 ± 15.8	326.8 ± 15.8	0.177
MCV, fL	86.2 ± 8.8	86.1 ± 8.9	0.995
ALT, U/L	20.0 (15.0, 27.0)	20.0 (15.0, 27.5)	0.818
AST, U/L	12.0 (8.0, 19.0)	12.0 (9.0, 18.0)	0.939
Albumin, g/L	34.8 ± 5.4	34.8 ± 5.5	0.953
Total bilirubin, umol/L	8.0 ± 2.4	7.8 ± 2.0	0.448
ALP, U/L	85.5 ± 15.7	85.4 ± 17.6	0.261
Glu, mmol/L	6.5 ± 1.7	6.2 ± 1.8	0.198
TG, mmol/L	1.8 ± 0.5	1.8 ± 0.4	0.779
TC, mmol/L	4.3 ± 1.1	4.3 ± 1.0	0.836
HDL-C, mmol/L	1.1 ± 0.3	1.1 ± 0.3	0.983
LDL-C, mmol/L	2.4 ± 0.7	2.4 ± 0.8	0.817
BUN, mol/L	16.8 (13.0, 20.6)	16.8 (13.3, 21.3)	0.627
Creatinine, μmol/L	587.0 (420.0, 801.0)	595.0 (449.0, 812.5)	0.290
Uric acid, μmol/L	372.0 (301.0, 450.0)	384.0 (310.0, 449.5)	0.309
PT, s	14.8 ± 4.0	14.6 ± 4.0	0.138
APTT, s	35.9 ± 4.0	35.8 ± 3.8	0.991
D dimer, ug/mL	1.8 (0.6, 4.2)	1.8 (0.6, 3.7)	0.834
INR	1.1 ± 0.2	1.1 ± 0.2	0.663
Sodium, mmol/L	139.3 ± 3.0	138.3 ± 3.0	0.921
Potassium, mmol/L	4.1 ± 0.7	4.0 ± 0.7	0.942
Calcium, mmol/L	2.2 ± 0.2	2.2 ± 0.3	0.592
Chloride, mmol/L	102.2 ± 4.5	102.2 ± 4.4	0.995
Phosphorus, mmol/L	1.4 ± 0.4	1.5 ± 0.3	0.600
TCO2, mmol/L	25.4 ± 3.8	25.3 ± 3.9	0.909
Ferritin, μg/L	133.0 (62.8, 271.0)	116.0 (53.1, 245.0)	0.051
iPTH, pg/mL	199.0 (91.4, 350.0)	200.8 (88.9, 343.4)	0.962
CRP, mg/L	3.8 (3.1, 11.2)	3.5 (3.1,11.0)	0.857
Primary outcome			
Death, *n* (%)	130 (12.5)	47 (10.6)	0.288

BMI: body mass index; CAPD: continuous ambulatory peritoneal dialysis; WBC: white blood cell; RBC: red blood cell; MPV: mean platelet volume; MCH: mean corpuscular hemoglobin; MCHC: mean corpuscular hemoglobin concentration; MCV: mean corpuscular volume; ALT: alanine aminotransferase; AST: aspartate aminotransferase; ALP: alkaline phosphatase; TG: triglyceride; TC: total cholesterol; HDL-C: high-density lipoprotein-cholesterol; LDL-C: low-density lipoprotein-cholesterol; BUN: blood urea nitrogen; PT: prothrombin time; APTT: activated partial thromboplastin time; INR: international normalized ratio; TCO_2_: total carbon dioxide; iPTH: intact parathyroid hormone; CRP: C-reactive protein

#### Construction of ML models in the training set

**Figure 3.**
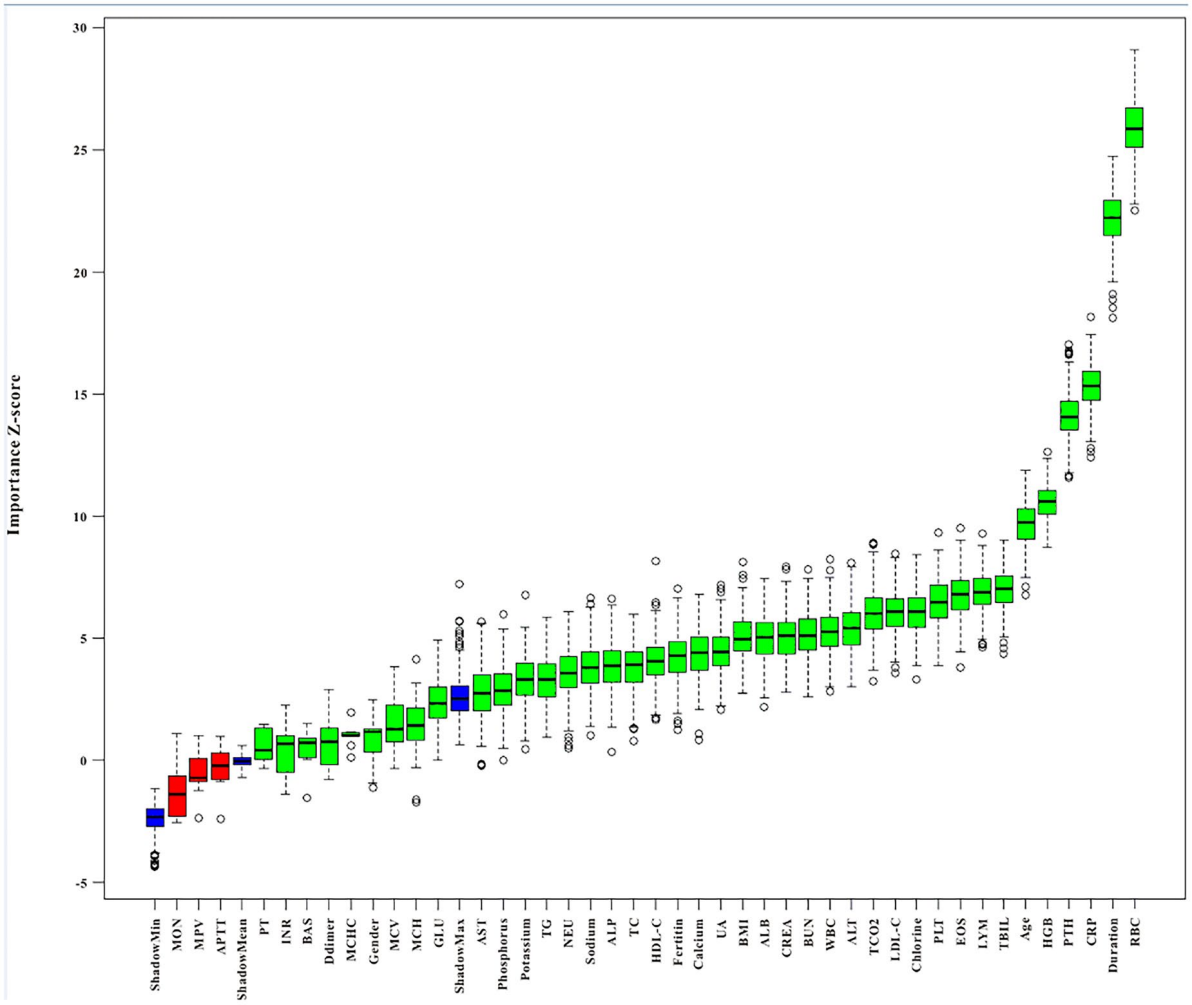
The feature importance based on Boruta feature selection method. MON, monocyte; MPV, mean platelet volume; APTT, activated partial thromboplastin time; PT, prothrombin time; INR, international normalized ratio; BAS, basophil; MCHC, mean corpuscular hemoglobin concentration; MCV, mean corpuscular volume; MCV, mean corpuscular volume; GLU, glucose; AST, aspartate aminotransferase; TG, triglyceride; NEU, neutrophil; ALP, alkaline phosphatase; TC, total cholesterol; HDL-C, high-density lipoprotein-cholesterol; UA, uric acid; BMI, body mass index; ALB, albumin; CREA, creatinine; BUN, blood urea nitrogen; WBC, white blood cell; ALT, alanine aminotransferase; TCO2, total carbon dioxide; LDL-C, low-density lipoproteincholesterol; PLT, platelet; EOS, eosinophil; LYM, lymphocyte; TBIL, total bilirubin; HGB, haemoglobin; PTH, intact parathyroid hormone; RBC, red blood cell.

Four demographic features and 38 blood indexes were initially included into this analysis. As shown in Figure S1, spearman correlation analysis determined that no clinical features have strong correlation with others. Subsequently, the Boruta feature selection method was employed to further select the important features of mortality. As displayed in Figure 3, three clinical features (monocyte, mean platelet volume, activated partial thromboplastin time) were identified as less important features, and thus were excluded.

**Figure 4.**
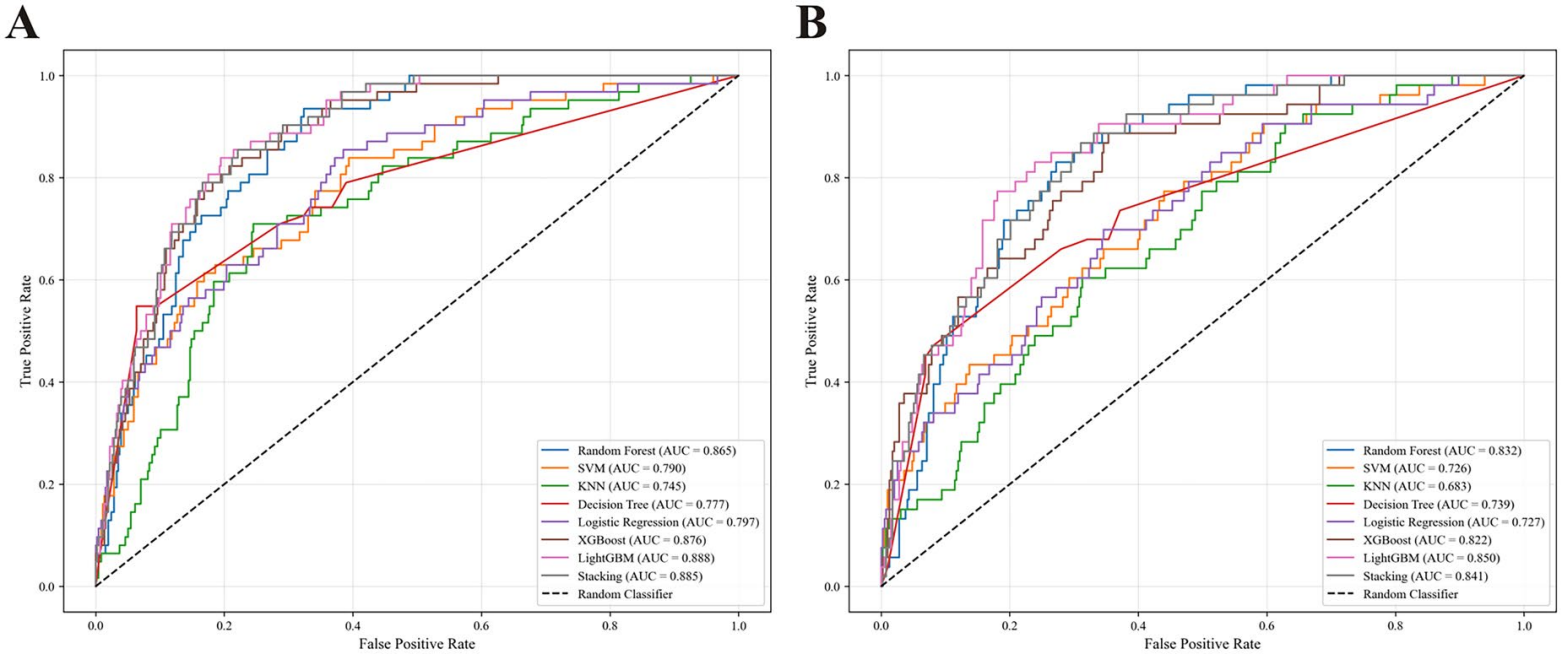
The ROC curves of nine machine learning models for the prediction of CAPD patients’ survival outcome in the internal validation cohort (A) and in the temporal validation cohort (B).

Eight ML technologies were utilized to selected the important clinical indexes and constructed the predictive models for 6-month mortality. The ROC curves of these models were displayed in Figure 4(A), respectively. The lightGBM algorithm obtained the best predictive performance (AUC:0.888, Figure 4A), and the RF model achieved the highest predictive accuracy (accuracy:0.882, Figure 4A) for the 6-month mortality of CAPD patients among the eight ML models. The other predictive parameters, such as specificity, sensitivity, and Brier score were exhibited in Table 2. Moreover, calibration curves, DCA curves and confusion matrices indicated that all eight models performed satisfactorily (Supplemental Figure 2, Supplemental Figure 4, and Supplemental Figure 6).

**Table 2.** Performance of the ML prediction models using all features.

Model	AUC (95%CI)	Accuracy	Sensitivity	Specificity	Brier score
Derivation cohort					
Logistic regression	0.80 (0.74–0.85)	0.77 (0.74–0.81)	0.70 (0.57–0.82)	0.57 (0.52–0.63)	0.16 (0.13–0.18)
Decision tree	0.78 (0.71–0.84)	0.71 (0.68–0.75)	0.55 (0.40–0.68)	0.84 (0.80–0.87)	0.17 (0.14–0.20)
Random forest	0.87 (0.82–0.90)	0.88 (0.85–0.91)	0.77 (0.66–0.89)	0.76 (0.72–0.80)	0.16 (0.15–0.17)
XGBoost	0.88 (0.83–0.91)	0.86 (0.83–0.90)	0.75 (0.67–0.87)	0.82 (0.78–0.86)	0.09 (0.08–0.12)
LightGBM	0.89 (0.85–0.92)	0.88 (0.85–0.91)	0.79 (0.69–0.92)	0.85 (0.79–0.90)	0.09 (0.07–0.11)
KNeighbors	0.75 (0.69–0.81)	0.37 (0.33–0.42)	0.64 (0.51–0.76)	0.59 (0.54–0.64)	0.33 (0.31–0.34)
SVM	0.79 (0.72–0.85)	0.77 (0.74–0.81)	0.69 (0.54–0.82)	0.65 (0.56–0.74)	0.15 (0.13–0.18)
Stacking model	0.88 (0.84–0.91)	0.87 (0.84–0.90)	0.72 (0.59–0.86)	0.82 (0.79–0.84)	0.10 (0.08–0.12)
Validation cohort					
Logistic regression	0.73 (0.65–0.80)	0.76 (0.71–0.79)	0.85 (0.76–0.94)	0.61 (0.56–0.65)	0.16 (0.13–0.18)
Decision tree	0.74 (0.66–0.81)	0.71 (0.67–0.76)	0.66 (0.53–0.78)	0.82 (0.78–0.85)	0.16 (0.14–0.20)
Random forest	0.83 (0.79–0.88)	0.85 (0.82–0.89)	0.79 (0.68–0.89)	0.75 (0.71–0.79)	0.15 (0.14–0.16)
XGBoost	0.82 (0.76–0.88)	0.87 (0.83–0.89)	0.79 (0.69–0.89)	0.81 (0.77–0.84)	0.10 (0.07–0.12)
LightGBM	0.85 (0.80–0.89)	0.87 (0.84–0.90)	0.89 (0.80–0.96)	0.76 (0.70–0.85)	0.09 (0.06–0.11)
KNeighbors	0.68 (0.61–0.75)	0.41 (0.36–0.45)	0.79 (0.68–0.89)	0.59 (0.54–0.63)	0.27 (0.25–0.29)
SVM	0.73 (0.66–0.80)	0.76 (0.72–0.80)	0.84 (0.74–0.92)	0.62 (0.57–0.66)	0.15 (0.13–0.18)
Stacking model	0.84 (0.79–0.89)	0.88 (0.84–0.91)	0.77 (0.67–0.88)	0.80 (0.75–0.86)	0.11 (0.09–0.13)

ML: machine learning; AUC: area under the curve of ROC; 95%CI: confidence interval; KNeighbors: k-Nearest Neighbor; XGBoost eXtreme Gradient Boosting; lightGBM: light gradient boosting machine; SVM support vector machine

#### ML model interpretations with SHAP

**Figure 5.**
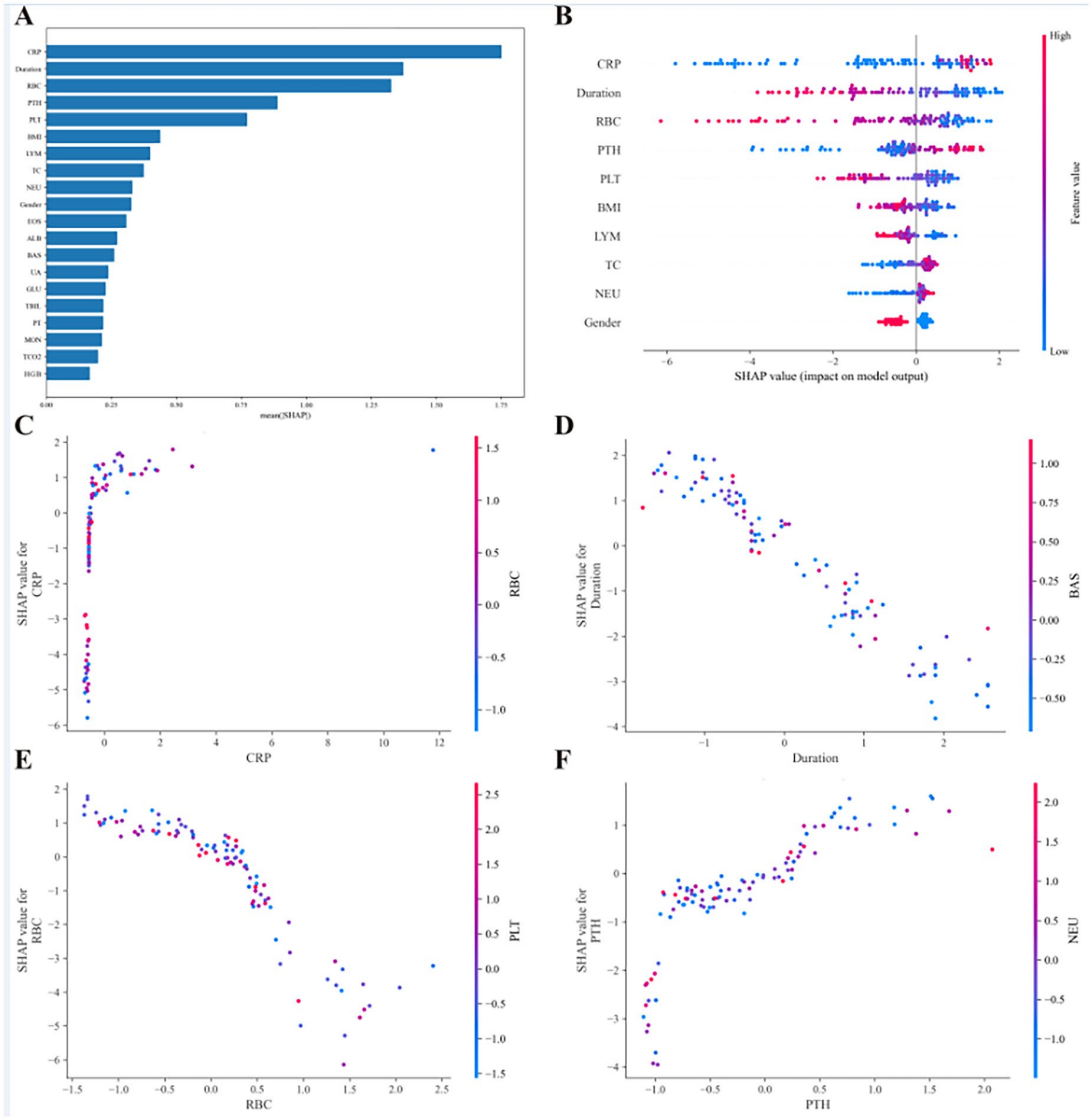
Global explanation for the lightGBM model by the SHAP analysis. The SHAP summary bar plot (A) and SHAP summary dot plot (B) display the feature importance in the lightGBM model. (C-F) SHAP dependence plots show the top four key features in the lightGBM model. CRP, Creactive protein; RBC, red blood cell; PTH, intact parathyroid hormone; PLT, platelet; BMI, body mass index; LYM, lymphocyte; TC, total cholesterol; NEU, neutrophil; ALB, albumin; BAS, basophil; UA, uric acid; CAPD, continuous ambulatory peritoneal dialysis; WBC, white blood cell; MPV, mean platelet volume; MCH, mean corpuscular haemoglobin; MCHC, mean corpuscular hemoglobin concentration; MCV, mean corpuscular volume; ALT, alanine aminotransferase; AST, aspartate aminotransferase; ALP, alkaline phosphatase; TG, triglyceride; HDL-C, high-density lipoproteincholesterol; LDL-C, low-density lipoprotein-cholesterol; BUN, blood urea nitrogen; PT, prothrombin time; APTT, activated partial thromboplastin time; INR, international normalized ratio; TCO2, total carbon dioxide; PTH, intact parathyroid hormone.

**Figure 6.**
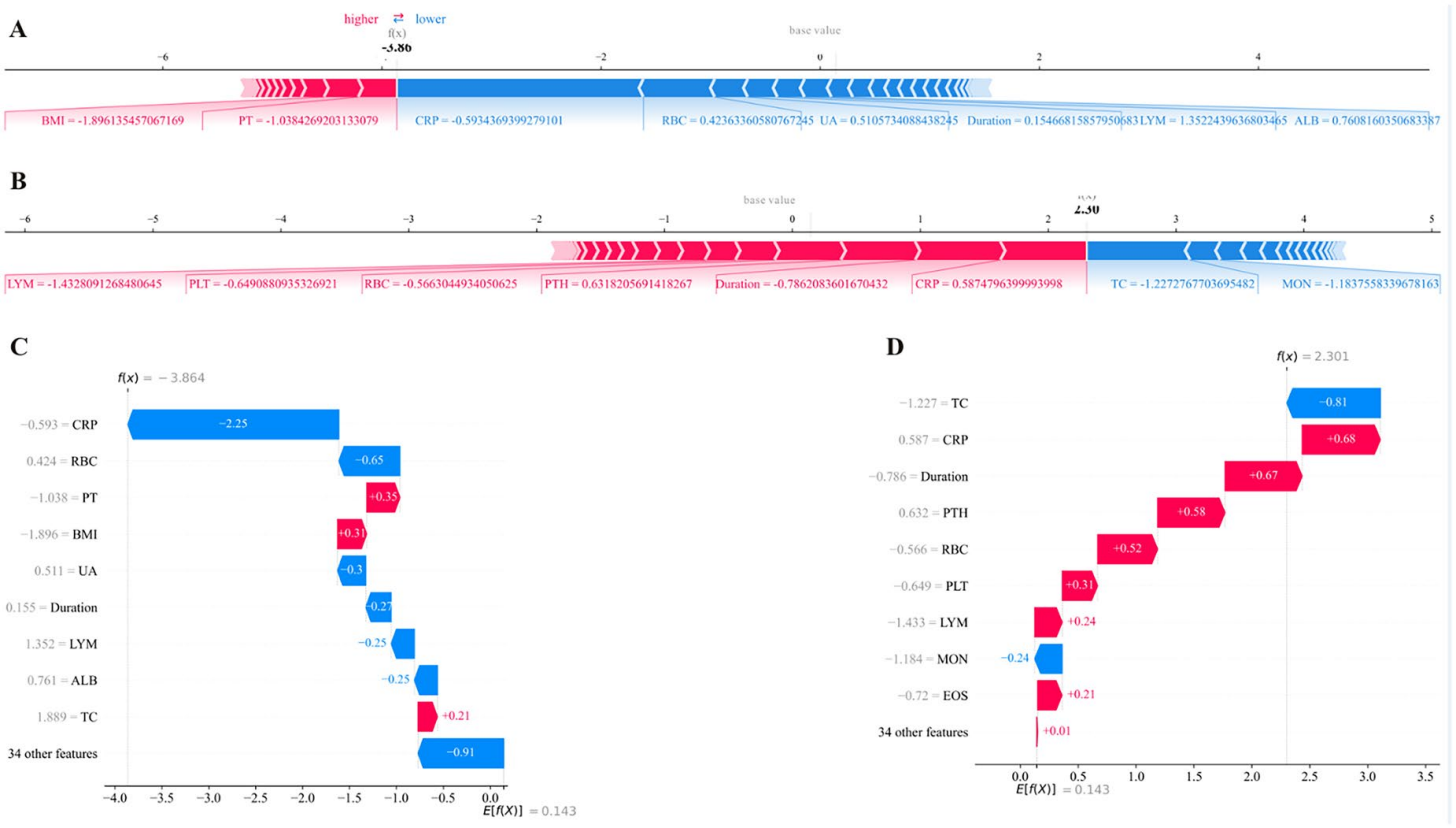
Local explanations of the lightGBM based on the SHAP force plots. SHAP force plots for the case who was alive (A, C) and the case who was died (B, D). BMI, body mass index; PT, prothrombin time; CRP, C-reactive protein; RBC, red blood cell; UA, uric acid; LYM, lymphocyte; ALB, albumin; PLT, platelet; PTH, intact parathyroid hormone; TC, total cholesterol; MON, monocyte.

We utilized the SHAP method to reasonably explain the lightGBM model. SHAP analysis could interpret the lightGBM model at the global and local levels. Global explanation described the overall functionality of the lightGBM model. The contributions of the clinical features to the predictive model were assessed using the average SHAP values (Figure 5A), and the top 10 clinical indexes were also displayed (Figure 5B). The top modifiable predictive features included C-reactive protein (CRP), serum albumin, hemoglobin, intact parathyroid hormone (iPTH), and red blood cell (RBC) count, all of which have established clinical relevance and actionable intervention targets in CAPD care. For local interpretation, SHAP dependence plots revealed how individual clinical features influence the prediction for the LightGBM model (Figure 5C–F). SHAP values less than 0 generally correspond to predictions of the negative class (low 6-month mortality risk). Furthermore, SHAP waterfall and force plots were used to explain individual predictions from the lightGBM model for representative patients who was died or alive within six months after routine visits (Figure 6A–D).

#### Real clinical application examples of the lightGBM model

**Figure 7.**
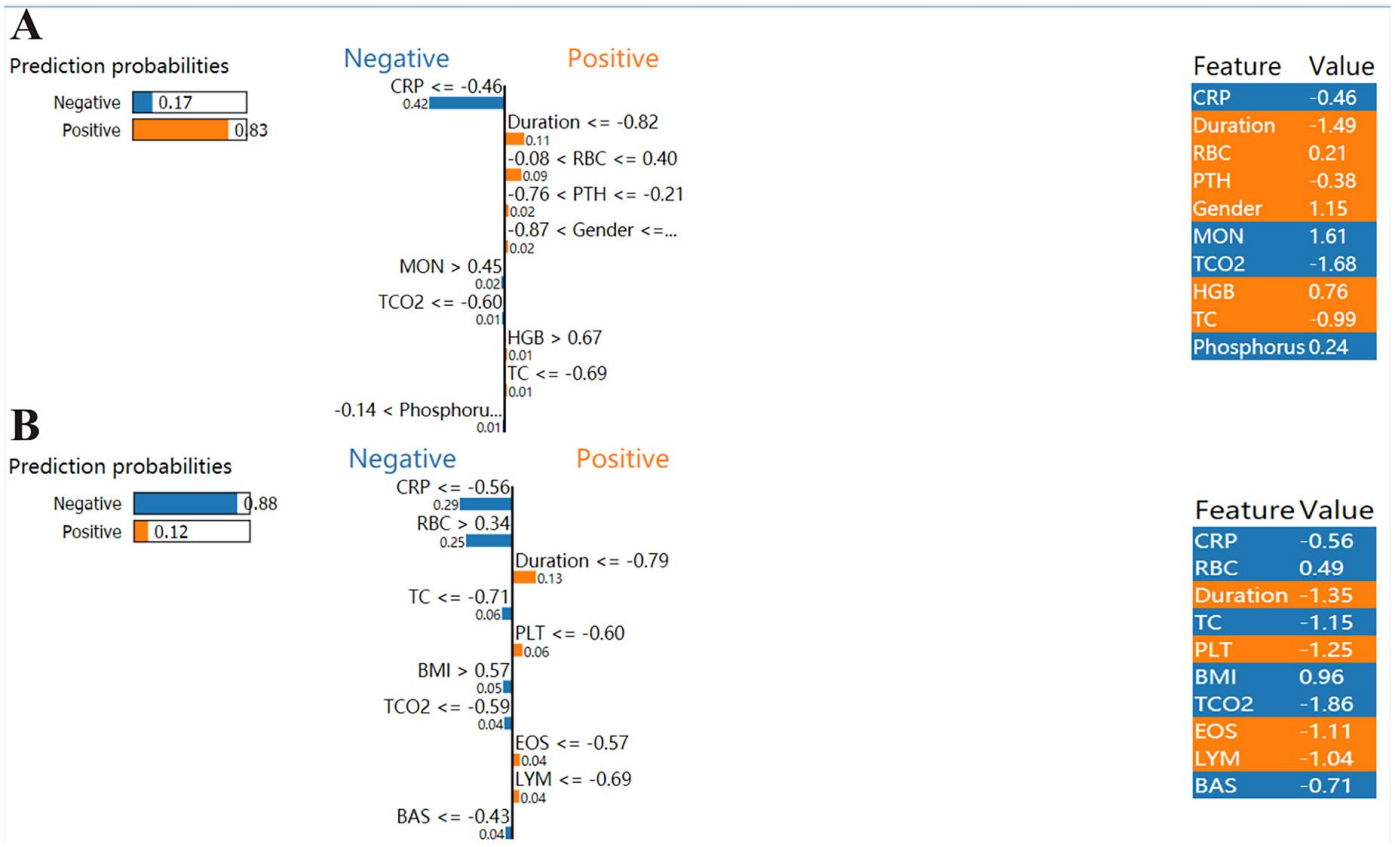
Local Interpretable of the lightGBM model based on the LIME method. LIME plots for the for the case who was died (A) and the case who was alive (B). CRP, Creactive protein; RBC, red blood cell; PTH, intact parathyroid hormone; MON, monocyte; TCO2, total carbon dioxide; HGB, haemoglobin; TC, total cholesterol; PLT, platelet; BMI, body mass index; EOS, eosinophil; LYM, lymphocyte; BAS, basophil.

For the better application of the lightGBM model in the clinical practice, the LIME method was used to show how the lightGBM model predicts the risk of 6-month death. A CAPD case was died within six months, who was correctly identified as high risk of death (the possibility was 0.83), mainly based on the following key features: low levels of duration, RBC and PTH, and high levels of HGB, while the low level of CRP had an incorrect effect on the final classification results (Figure 7A). We randomly selected a CAPD patient who was alive after six months of routine visits. The LIME method correctly predicted a low risk of death (the possibility was 0.12), mainly based on the following key features: low levels of CRP, TC, and high levels of RBC and BMI, while the low levels of duration and platelet (Figure 7B). In clinical practice, conflicting indicators make making definitive decisions challenging. Therefore, local interpretation using the LIME algorithm has significant clinical value for aiding clinicians in assessing the risk of 6-month mortality for CAPD patients.

#### Temporal validation the ML models

In the temporal validation cohort, the ROC curves of the eight ML models were displayed in Figure 4(B). The lightGBM algorithm still obtained the best predictive performance in this cohort (AUC:0.850, Figure 4B), while the Stacking model achieved highest predictive accuracy (accuracy:0.877, Figure 4B) in the temporal validation cohort. The other predictive parameters were also exhibited in Table 2. Moreover, calibration curves, DCA curves and confusion matrices indicated that all eight models performed satisfactorily in the temporal validation cohort (Supplemental Figure 3 and Supplemental Figure 5). Moreover, our model achieves comparable discriminative performance relative to recently existing studies, while using only time-updated routine laboratory data with no specialized imaging or testing required (Supplemental Table 3).

## Discussion

This retrospective study firstly resorted eight advanced ML models for the 6-month mortality in CAPD patients using routinely available and time-updated EHR data and demonstrated that the excellent predictive ability of the lightGBM model in both internal validation and temporal validation cohorts, highlighting the robustness and universality of the EHR data-based model. Importantly, the SHAP method provides transparent explanation of the lightGBM model. In brief, these results provide an innovative strategy for the convenient and accurate prediction of survival outcome of CAPD patients, emphasizing the importance of routinely available and time-updated EHR data in combination with ML techniques.

Although substantial progress has been made in medical knowledge and healthcare infrastructure in past years, the clinical outlook for patients undergoing CAPD has remained stubbornly poor. Therefore, the timely risk stratification of CAPD patients is a critical step toward enabling targeted therapies and improving clinical outcomes. Despite numerous research efforts to develop robust prognostic tools, a significant translational gap persists, and no specific method has achieved widespread clinical adoption. Yan et al. enrolled 316 CAPD patients retrospectively and established a novel risk score based on the aggregate index of systemic inflammation, identifying an independent association with cardiovascular risk for CAPD patients [19]. Another study also included 161 patients with peritoneal dialysis-associated peritonitis and developed a risk score based on the serum biomarkers, average residual urine volume and urea clearance rate and demonstrated that this risk score achieved a sensitivity of 88.5% and a specificity of 74.3% with an AUC of 0.895 (95%CI 0.847–0.943) for the risk of treatment failure in these individuals [20]. In addition, Wan et al. also constructed a nomogram based on the initial transthoracic echocardiography score and clinical data from 274 CAPD patients and gained an AUC of 0.830 for the risk of 5-year all-cause mortality [21]. While these models show promising accuracy, their translational potential is hampered by a ‘specialized-but-inaccessible’ paradox; they depend on resources or expertise not available in routine, resource-variable clinical environments. This work addresses this gap by championing an ‘accessible-and-actionable’ paradigm. Our model capitalizes on the dense, longitudinal data generated from mandatory quarterly/monthly lab tests, transforming routine EHR data into a powerful prognostic tool. By using only universally available variables, our strategy ensures broad applicability without additional costs or procedures that supports broad scalability across all clinical settings, from large academic centers to community dialysis clinics. We envision this ML model being deployed pervasively across HD facilities for instant risk profiling, effectively triaging patients for escalated care and potentially optimizing resource allocation and outcomes.

Artificial intelligence, particularly through ML and deep learning, has advanced the analysis of multi-omics data and prognostic model development, thereby improving precise diagnosis and outcome prediction for CAPD patients. Xu et al. retrospectively analyzed 1,006 CAPD patients and demonstrated that ML models outperformed conventional Cox regression in discriminating patient risk using readily available clinical, laboratory, and electrocardiographic variables [22]. Similar superiority of ML approaches has been corroborated by other recent studies focusing on longer-term outcomes in this population [23,24]. However, a common limitation among these prior ML efforts is their reliance on static modeling frameworks, which utilize baseline or fixed-timepoint measurements. While informative, such models operate under the assumption that risk factors remain constant over time-an assumption often inconsistent with the dynamic clinical trajectory of CAPD patients. In contrast, our study, along with another recent ML investigation [25], challenges this static paradigm by incorporating a time series clinical data structure. This methodology recognizes that patient risk is fluid and continuously influenced by the most recent laboratory results. The strong performance of our model, particularly its robustness in temporal validation, underscores the critical importance of capturing such dynamism. More importantly, to translate the predictive outputs of our time-updated early-warning system into actionable clinical practice for CAPD patients, we established a standardized risk-stratified intervention framework directly anchored to the model’s 6-month all-cause mortality risk estimates and key modifiable predictive features identified *via* SHAP: for low-risk patients with a predicted mortality risk <5%, we recommend continuation of standard quarterly CAPD follow-up, routine laboratory monitoring, and guideline-concordant management of ESKD. For moderate-risk patients with a predicted risk of 5%–20%, we trigger personalized, feature-specific interventions targeting the identified factors, including renal dietitian-need nutritional assessment and dietary optimization for those with hypoalbuminemia or mineral metabolism disorders, evidence-based correction of anemia, plus mandatory monthly laboratory follow-up to track intervention response and dynamic risk changes. For high-risk patients with a predicted risk >20%, we mandate urgent multidisciplinary care escalation, including immediate medical consultation for cardiorenal status, goals-of-care discussions with patients and families, and weekly to bi-weekly clinical and laboratory monitoring with dynamic adjustment of dialysis prescriptions and medical regimens. This integrated framework provides frontline clinicians with a clear, workflow-aligned roadmap for embedding the predictive model into routine CAPD care, ensuring the model’s risk stratification outputs directly translate into optimized, individualized patient management.

The interpretation of our findings must be tempered by an acknowledgment of certain constraints inherent to the study design. As a retrospective analysis conducted within a single regional cohort, our study may be susceptible to selection bias and unmeasured confounding, as several prognostically relevant variables including quantified frailty, functional status, medication adherence, health literacy, and socioeconomic factors were not available in our dataset. Furthermore, while our analysis identifies significant predictors, it remains associational and does not provide mechanistic insight or establish causality. Additionally, although our approach utilized time-updated data, it was based on the most recent clinical assessments rather than explicitly modeling the dynamic trajectories of biomarkers over time. These limitations directly inform future research priorities. There is a clear need for prospective, multi-center external validation across diverse populations to verify generalizability. Moreover, impact studies are essential to determine whether the clinical implementation of this model within EHR systems, coupled with decision support, actually improves patient outcomes (such as reducing hospitalizations), facilitates goal-concordant care, or optimizes healthcare resource allocation.

Future studies should prioritize prospective, multi-center validation of ML models across diverse geographic and demographic CAPD populations to establish generalizability and reduce center-specific bias. Methodologically, moving beyond static or last-observation approaches toward explicit modeling of longitudinal biomarker trajectories, using time-series, recurrent, or transformer-based architectures, may better capture dynamic risk evolution and improve near-term prognostication. Embedding models within EHR systems with real-time updating, calibrated risk thresholds, and clinician-facing explanations warrants pragmatic impact studies to determine effects on clinical decision-making, hospitalization rates, goal-concordant care, and healthcare resource allocation. Finally, hybrid frameworks that combine causal inference with explainable machine learning may help distinguish modifiable risk factors from correlates, thereby enabling intervention-focused decision support rather than prediction alone.

In summary, our time-updated, explainable ML framework is not limited to CAPD patients, and can serve as a scalable template for risk stratification in other chronic disease populations, including non-dialysis CKD, heart failure, diabetes, and chronic obstructive pulmonary disease. Our approach-using routinely collected EHR data, rigorous cluster-aware validation, and transparent, intervention-focused explainability can be readily adapted to any chronic condition where dynamic risk monitoring is critical to improving patient outcomes, extending the relevance of this work beyond nephrology.

## Supplementary Material

Supplemental_materials (1).docx

## Data Availability

The data that support the findings of this study are available from the corresponding author, ZYM, upon reasonable request.
