# Supplementary material for: Time-updated explainable machine learning predicts short-term mortality in peritoneal dialysis patients
Source: Ren Fail. 2026 May 27;48(1):2666955. doi: 10.1080/0886022X.2026.2666955 (PMC13220578; doi:10.1080/0886022X.2026.2666955)
Supplement: Supplemental_materials (1).docx [file IRNF_A_2666955_SM5125.docx]

**Supplemental Methods**

**Data Splitting Framework:** The splitting strategy is tailored to preserve observation independence, mitigate data leakage, and accommodate patient overlap in the temporal validation cohort.

As for the derivation cohort, we firstly partitioned into the training (50%) and internal validation (50%) subsets using a modified stratified patient-level clustering approach: stratify patients by “event status percentiles” (the proportion of their observations with 6-month mortality) to ensure balanced distribution of patients across subsets. Secondly, cluster all independent observations from the same patient into a single subset (training or internal validation) to avoid “patient-specific information leakage” (the model learning individual idiosyncrasies from one subset and testing on the same patient’s observations in another subset). Finally, model training and hyperparameter tuning were performed on the training set using repeated 5-fold cluster cross-validation, further reinforcing protection against within-patient correlation.

As for the temporal validation cohort, this cohort includes two patient subgroups: overlapping patients (n=189, patients with prior observations in the derivation cohort), and new patients (n=257, patients with no prior observations in the derivation cohort). And to ensure validity despite patient overlap, we conducted two strategies: time-blocked observation isolation (For overlapping patients, only new observations collected in 2024-2025 are included in the temporal cohort, with outcome windows strictly following these 2024-2025 tests. No observations or outcome windows from the 2021–2023 period are reused or cross-referenced) and no backward feature leakage (All features for temporal cohort observations are restricted to data collected at the time of the 2024-2025 test. No retrospective data from the 2021-2023 period are incorporated to augment 2024-2025 observations).

**Data leakage mitigation safeguards:** Three layers of safeguards ensure no artificial performance inflation.

Patient-level clustering within time blocks: Observations from the same patient are clustered within their respective time cohorts (2021-2023 or 2024-2025) but never across time blocks;

Non-overlapping outcome windows: Outcome windows for derivation and temporal cohorts are mutually exclusive (derivation outcomes end by 2023-2024; temporal outcomes start in 2024-2025);

Blinded feature engineering: Feature selection (two-stage filtering + Boruta) and model training were conducted exclusively on the derivation cohort, with no access to temporal validation cohort data during model development.

**Sensitivity analysis for patient overlap:** To verify no bias from overlapping patients, we compared the performance of the optimal LightGBM model between overlapping and new patients in the temporal validation cohort (Table S2). All data align with the previously reported results (ΔAUC=0.015, p=0.689) and confirm no performance bias from patient overlap. This consistency proves the model does not "memorize" overlapping patients, which means that its predictions are driven by current clinical parameters, not prior patient-specific data.

**Machine Learning (ML) Models:** ML models are well-suited for clinical analysis tasks due to their flexibility, as they require fewer prior assumptions regarding data distributions and variable relationships. Their capability to handle numerous potential covariates and identify complex patterns makes them effective for developing optimized risk models. Accordingly, we applied nine distinct ML algorithms: Logistic Regression, Random Forest, k-Nearest Neighbors, Decision Tree, eXtreme Gradient Boosting, Light Gradient Boosting Machine, Support Vector Machine, and a Stacking Ensemble Classifier. These methods were selected based on their established applicability to similar data types and tasks.

**Feature Engineering:** SHAP analysis was employed to identify potentially significant predictor variables. We investigated whether model performance could be maintained or improved using reduced feature sets. During model development, features with the smallest impact on the model output were iteratively removed, and the models were refitted with the remaining features. This recursive elimination process continued until a notable decline in performance was observed. Additionally, SHAP analysis helped determine optimal clinical thresholds for specific continuous variables, facilitating model simplification for future application and refining the models by focusing on the most predictive features.

**Statistical Analysis:** All analyses were conducted using Python (Version 3.11) and R (version 4.3.2). The specific libraries utilized for various analytical steps are listed below:

- **Data Preprocessing and Visualization:** numpy, pandas, matplotlib, seaborn, sklearn.preprocessing (MinMaxScaler, StandardScaler, OrdinalEncoder, OneHotEncoder).
- **Model Development and Evaluation:** sklearn.model_selection (RandomizedSearchCV, train_test_split, cross_val_score), sklearn.ensemble (RandomForestClassifier, StackingClassifier), sklearn.svm (SVC), sklearn.linear_model (LogisticRegression), sklearn.tree (DecisionTreeRegressor), sklearn.neighbors (KNeighborsClassifier), xgboost, lightgbm, sklearn.naive_bayes (GaussianNB), shap, sklearn.metrics (roc_curve, auc, roc_auc_score, f1_score, confusion_matrix, brier_score_loss), sklearn.calibration (calibration_curve).


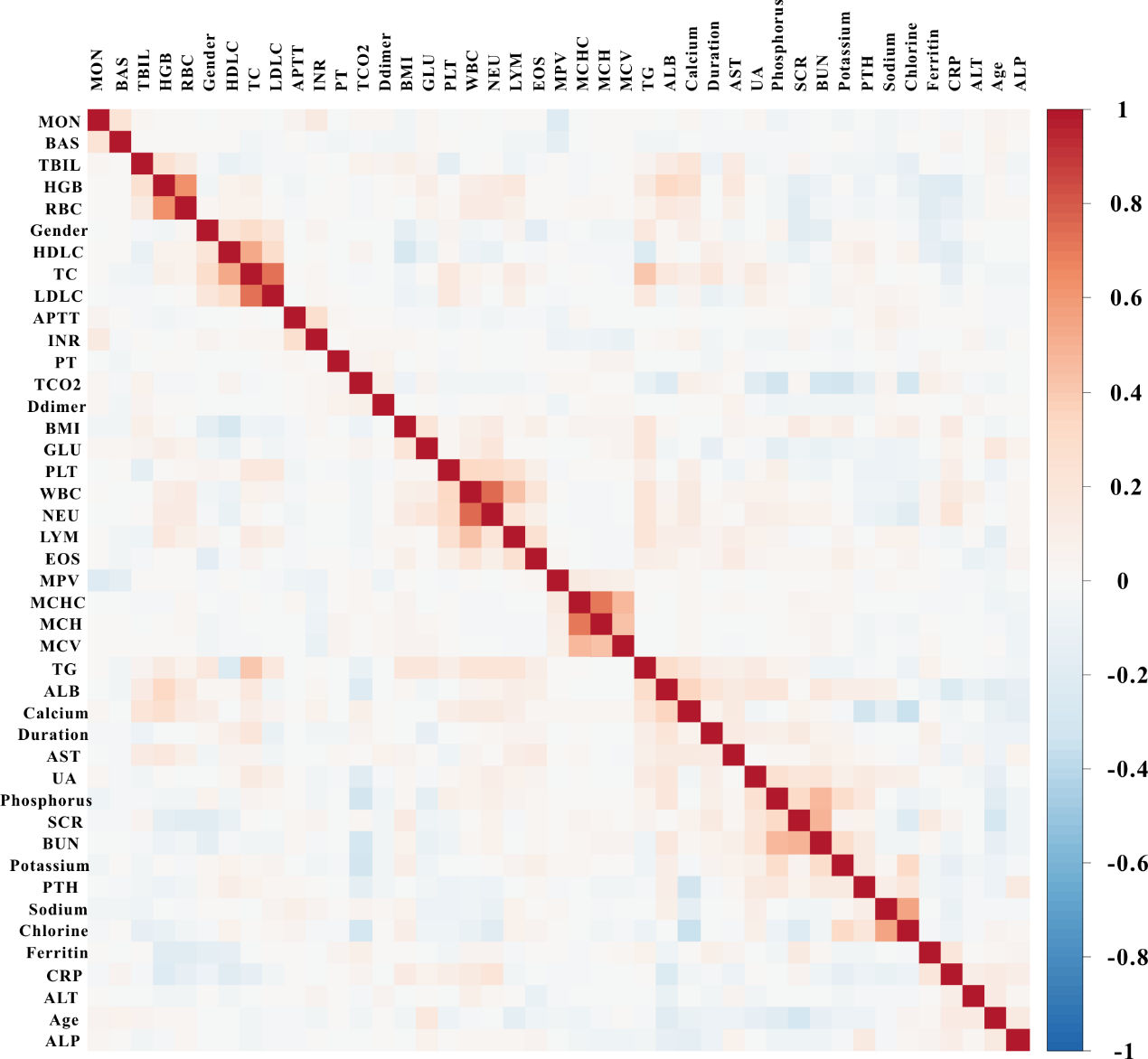


**Supplemental Figure 1** **The correlation heatmap plot of all features.**


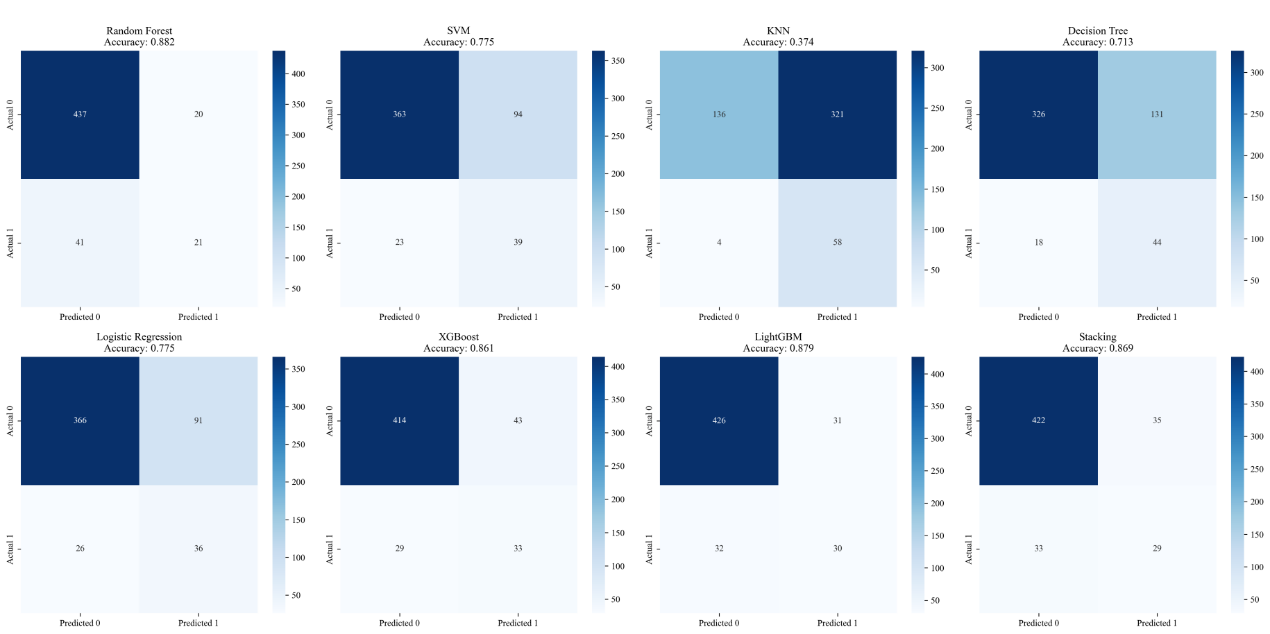


**Supplementary Figure 2. The confusion Matrix for 6-month mortality risk stratification models with different ML algorithms in the internal validation**. SVM: Support Vector machine; KNN: k-Nearest Neighbor; XGBoost: eXtreme Gradient Boosting; lightGBM: Light Gradient Boosting Machine.


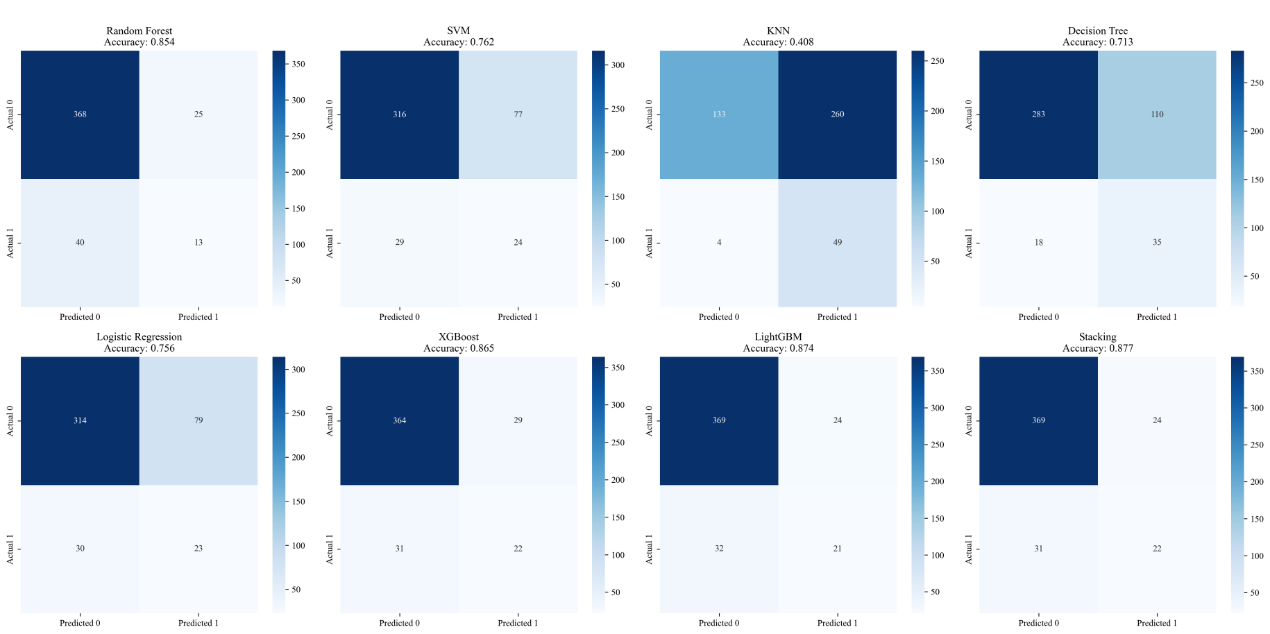


**Supplementary Figure 3.** **The confusion Matrix for 6-month mortality risk stratification models with different ML algorithms in the temporal validation**. SVM: Support Vector machine; KNN: k-Nearest Neighbor; XGBoost: eXtreme Gradient Boosting; lightGBM: Light Gradient Boosting Machine.

**
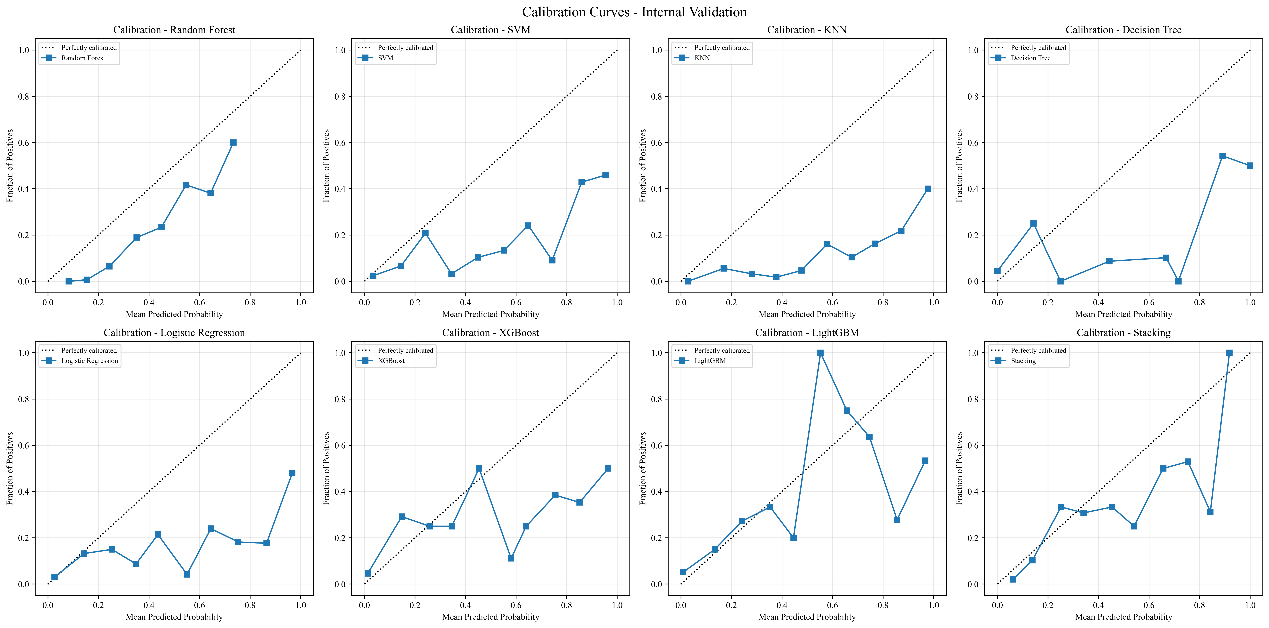
**

**Supplementary Figure 4.** **The calibration plot for 6-month mortality risk stratification models with different ML algorithms in the internal validation**. SVM: Support Vector machine; KNN: k-Nearest Neighbor; XGBoost: eXtreme Gradient Boosting; lightGBM: Light Gradient Boosting Machine.


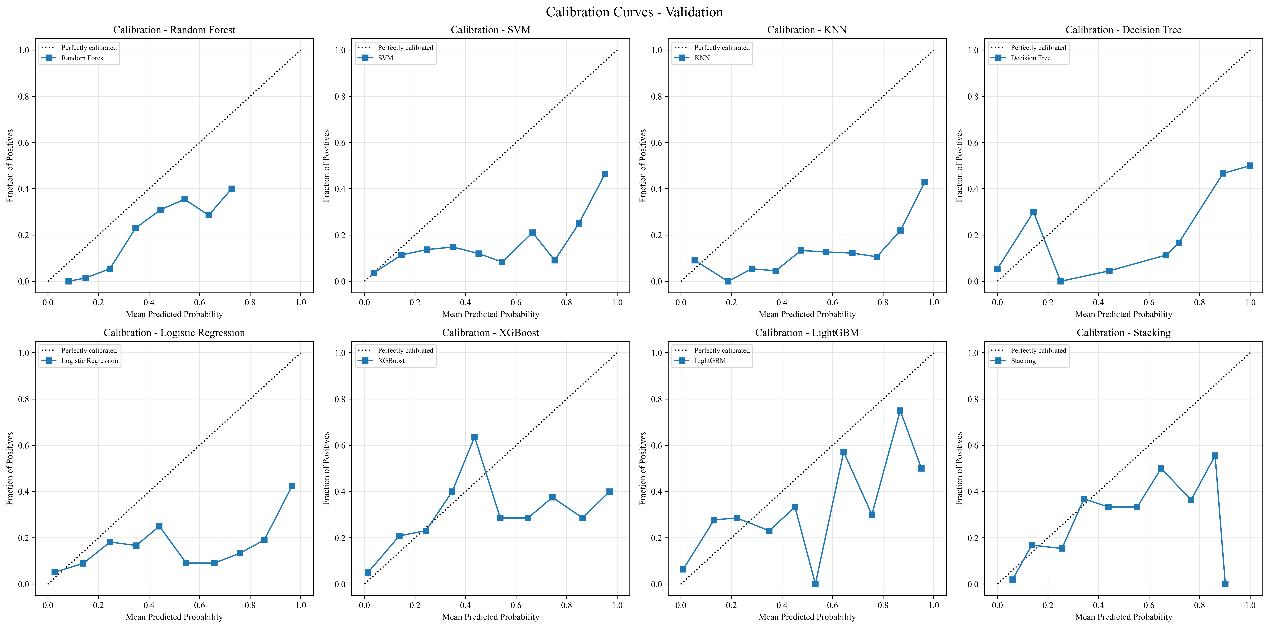


**Supplementary Figure 5.** **The calibration plot for 6-month mortality risk stratification models with different ML algorithms in the temporal validation**. SVM: Support Vector machine; KNN: k-Nearest Neighbor; XGBoost: eXtreme Gradient Boosting; lightGBM: Light Gradient Boosting Machine.


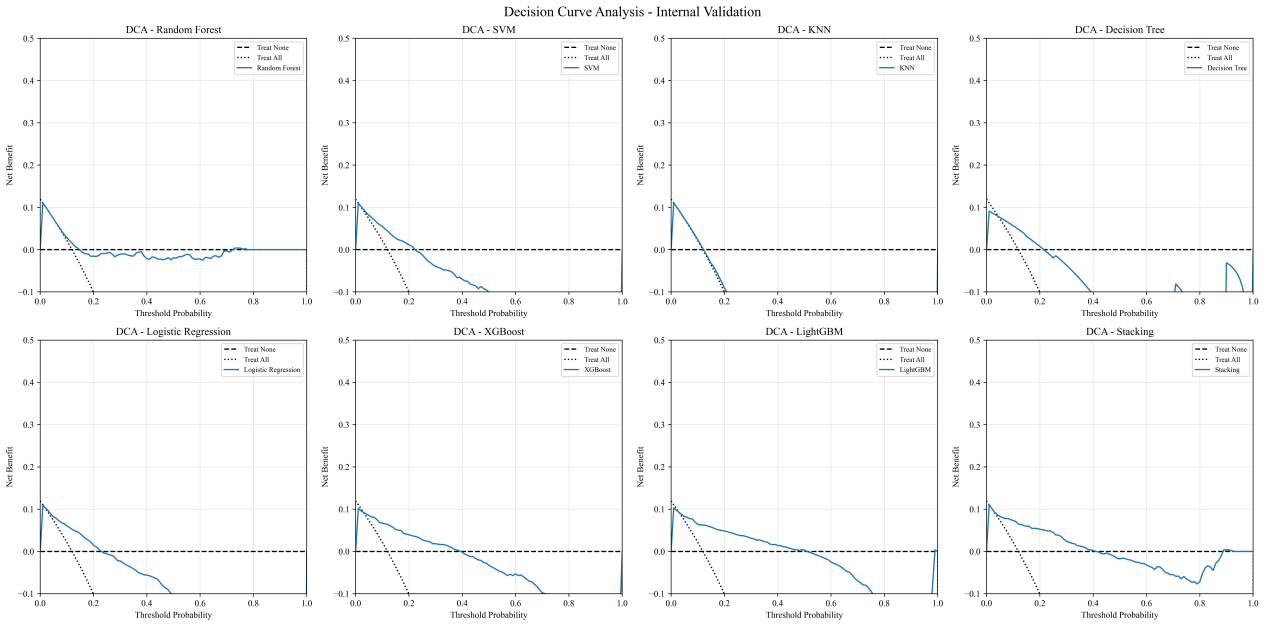


**Supplementary Figure 6.** **The decision curve analysis plots for 6-month mortality risk stratification models with different ML algorithms in the internal validation**. SVM: Support Vector machine; KNN: k-Nearest Neighbor; XGBoost: eXtreme Gradient Boosting; lightGBM: Light Gradient Boosting Machine.


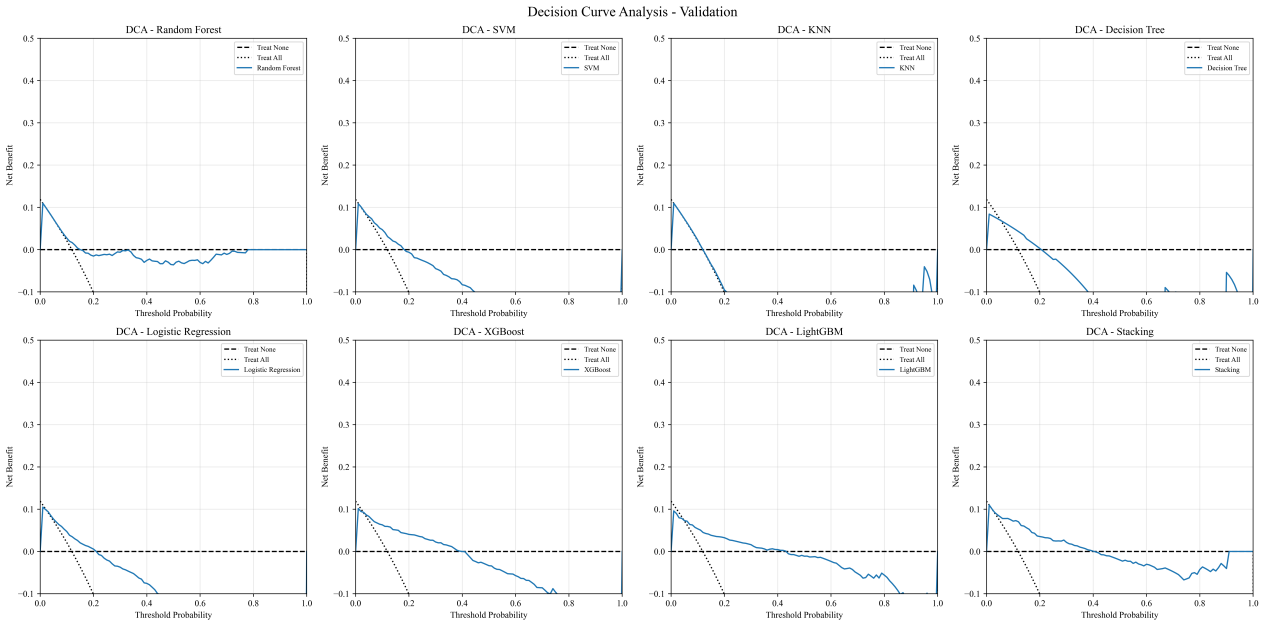


**Supplementary Figure 7.** **The decision curve analysis plots for 6-month mortality risk stratification models with different ML algorithms in the temporal validation**. SVM: Support Vector machine; KNN: k-Nearest Neighbor; XGBoost: eXtreme Gradient Boosting; lightGBM: Light Gradient Boosting Machine.

**Supplementary Table 1 Detailed information of the enrolled 43 features in the original feature set**

| **Feature source** | **Feature name** | **Feature type** |
| --- | --- | --- |
| **Sociodemographic information** |  |  |
|  | Gender | Categorical feature |
|  | Age | Continuous feature |
|  | BMI | Continuous feature |
| **Personal history** |  |  |
|  | Duration of CAPD | Continuous feature |
| **Laboratory test indexes** |  |  |
|  | WBC (Routine blood test) | Continuous feature |
|  | Neutrophils (Routine blood test) | Continuous feature |
|  | Lymphocytes (Routine blood test) | Continuous feature |
|  | Monocytes (Routine blood test) | Continuous feature |
|  | Eosinophiles (Routine blood test) | Continuous feature |
|  | PLT (Routine blood test) | Continuous feature |
|  | Hemoglobin (Routine blood test) | Continuous feature |
|  | CRP (Routine blood test) | Continuous feature |
|  | RBC (Routine blood test) | Continuous feature |
|  | MCV (Routine blood test) | Continuous feature |
|  | MCH (Routine blood test) | Continuous feature |
|  | MCHC (Routine blood test) | Continuous feature |
|  | MPV (Routine blood test) | Continuous feature |
|  | ALT (Biochemical test) | Continuous feature |
|  | AST (Biochemical test) | Continuous feature |
|  | TBil (Biochemical test) | Continuous feature |
|  | Albumin (Biochemical test) | Continuous feature |
|  | ALP (Biochemical test) | Continuous feature |
|  | TG (Biochemical test) | Continuous feature |
|  | Total cholesterol (Biochemical test) | Continuous feature |
|  | HDL-C (Biochemical test) | Continuous feature |
|  | LDL-C (Biochemical test) | Continuous feature |
|  | Blood glucose (Biochemical test) | Continuous feature |
|  | BUN (Biochemical test) | Categorical feature |
|  | Creatinine (Biochemical test) | Continuous feature |
|  | Uric acid (Biochemical test) | Continuous feature |
|  | PT (coagulation test) | Continuous feature |
|  | APTT(coagulation test) | Continuous feature |
|  | D dimer(coagulation test) | Categorical feature |
|  | INR(coagulation test) | Continuous feature |
|  | Sodium (Biochemical test) | Continuous feature |
|  | Potassium (Biochemical test) | Continuous feature |
|  | Calcium (Biochemical test) | Continuous feature |
|  | Chlorine (Biochemical test) | Categorical feature |
|  | Phosphorus (Biochemical test) | Continuous feature |
|  | TCO2 (Biochemical test) | Continuous feature |
|  | Ferritin (Biochemical test) | Continuous feature |
|  | iPTH (Biochemical test) | Continuous feature |
|  | CRP (Biochemical test) | Categorical feature |

BMI, body mass index, CAPD, continuous ambulatory peritoneal dialysis, WBC, white blood cell, RBC, red blood cell, MPV, mean platelet volume, MCH, mean corpuscular hemoglobin, MCHC, mean corpuscular hemoglobin concentration, MCV, mean corpuscular volume, ALT, alanine aminotransferase, AST, aspartate aminotransferase, ALP, alkaline phosphatase, TG, triglyceride, TC, total cholesterol, HDL-C, high-density lipoprotein-cholesterol, LDL-C, low-density lipoprotein-cholesterol, BUN, blood urea nitrogen, PT, prothrombin time, APTT, activated partial thromboplastin time, INR, international normalized ratio, TCO2, total carbon dioxide, iPTH, intact parathyroid hormone, CRP, C-reactive protein.

**Table S2 The performance of lightGBM model in the temporal validation cohort**

| Performance metric | Overlapping patients (n=189) | New patients (n=257) | P value^1^ |
| --- | --- | --- | --- |
| AUC (95% CI) | 0.842 (0.785–0.899) | 0.857 (0.808–0.906) | 0.689 |
| Accuracy (95% CI) | 0.865 (0.812–0.918) | 0.879 (0.835–0.923) | 0.614 |
| Sensitivity (95% CI) | 0.826 (0.719–0.933) | 0.841 (0.753–0.929) | 0.792 |
| Specificity (95% CI) | 0.783 (0.668-0.898) | 0.802 (0.715-0.889) | 0.745 |
| Brier Score (95% CI) | 0.098 (0.096–0.100) | 0.092 (0.091–0.093) | 0.063 |

^1^Statistical comparisons: Two-sided z-test for AUC; chi-square test for accuracy, sensitivity, and specificity and the independent samples t-test for Brier score.

**Table S3 The comparison of recently published ML models for CAPD patients**

| Study | Sample Size | Time Horizon | Model Type | AUC/C-index | Key Predictors |
| --- | --- | --- | --- | --- | --- |
| Xu et al. 2024 (1) | 3880 (multi-centre, China) | 3-month mortality | CVDformer | 0.88 | Clinical, laboratory, and dialysis data |
| Ali et al. 2025 (2) | 27111 (multi-centre, UK) | All-cause mortality | XGBoost | 0.83 | Clinical, and laboratory characteristics |
| Lin et al. 2025 (3) | 666 (single centre, China) | All-cause mortality | Random forest | 0.89 | Blood pressure variability, clinical, and laboratory characteristics |
| Lee et al. 2025 (4) | 132 (single centre, China) |  | Support vector machine | 0.87 | Peritoneal equilibration test, clinical, and laboratory characteristics |
| Our Model | 1484 (single centre, China) | 6-month mortality | LightGBM | 0.89 | Dynamic clinical, and laboratory characteristics |

**References:**

1. Xu X, Xu Z, Ma T, et al. Machine learning for identification of short-term all-cause and cardiovascular deaths among patients undergoing peritoneal dialysis. *Clin Kidney J*. 2024;17(9):sfae242.

1. Ali H, Casula AM, Paola A, et al. "PD-PREDICT": A Machine Learning Model for Patient Survival in Peritoneal Dialysis. *ASAIO J*.
2. Lin Y, Yi C, Cao P, et al. Visit-to-visit blood pressure variability and clinical outcomes in peritoneal dialysis - based on machine learning algorithms. *Hypertens Res*. 2025;48(5):1702-1715.
3. Lee CC, Chao JY, Liu KH, et al. Longitudinal Changes in Peritoneal Transport and Their Impact on Dialysis Outcomes: A Machine Learning Approach Integrating Clinical and Biomarker Data. *Am J Nephrol*. 2025;56(6):688-701.
